# Supplementary figures and images for: The 5′-3′ Exoribonuclease XRN4 Regulates Auxin Response via the Degradation of Auxin Receptor Transcripts
Source: Genes (Basel). 2018 Dec 17;9(12):638. doi: 10.3390/genes9120638 (PMC6316084; doi:10.3390/genes9120638)

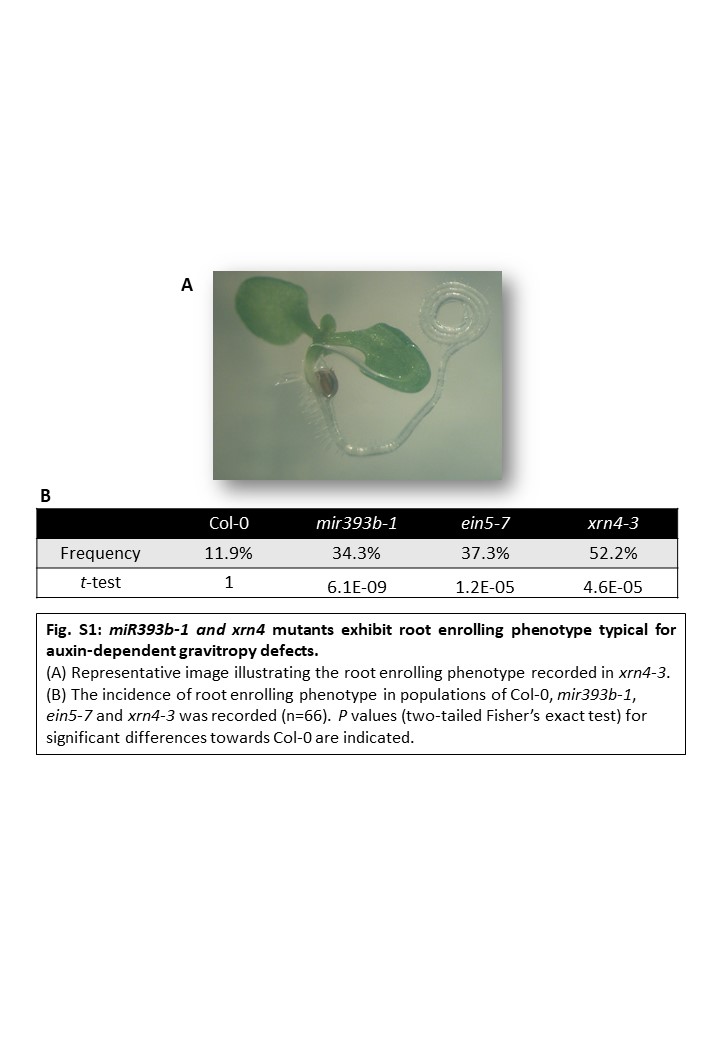

Supplement: Supplementary file 1 [file genes-09-00638-s001.zip › SF1.jpg]

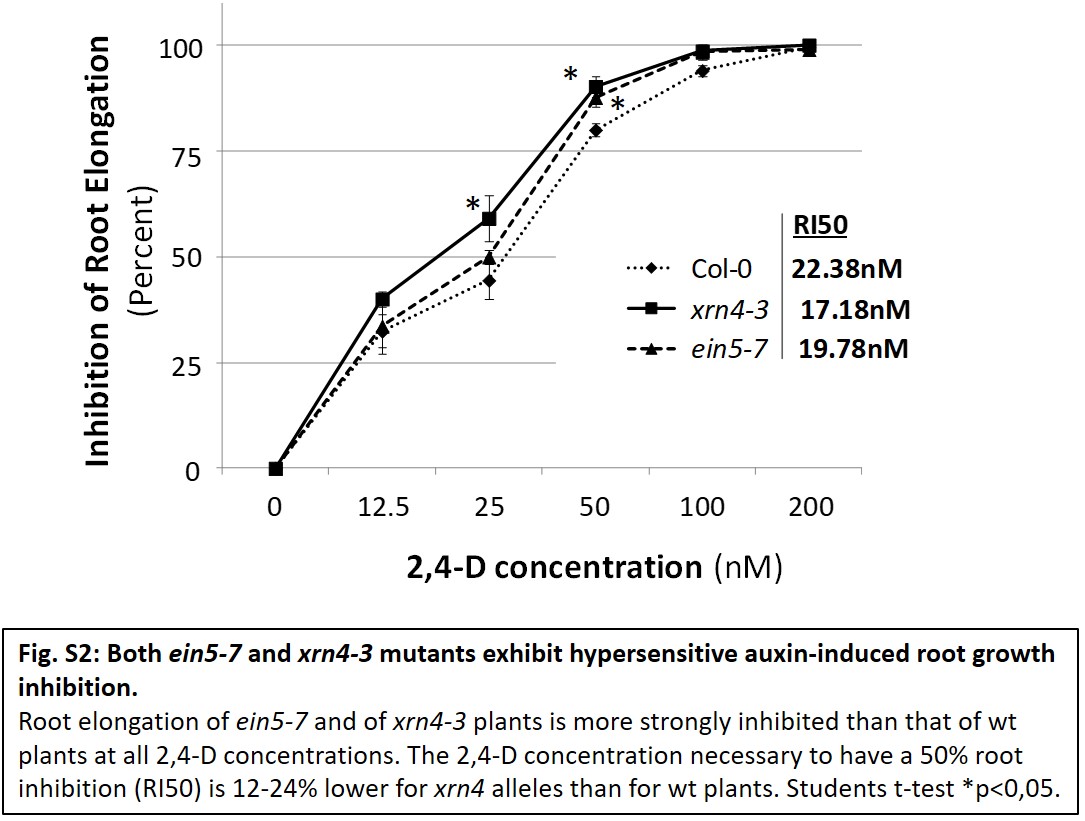

Supplement: Supplementary file 1 [file genes-09-00638-s001.zip › SF2.jpg]

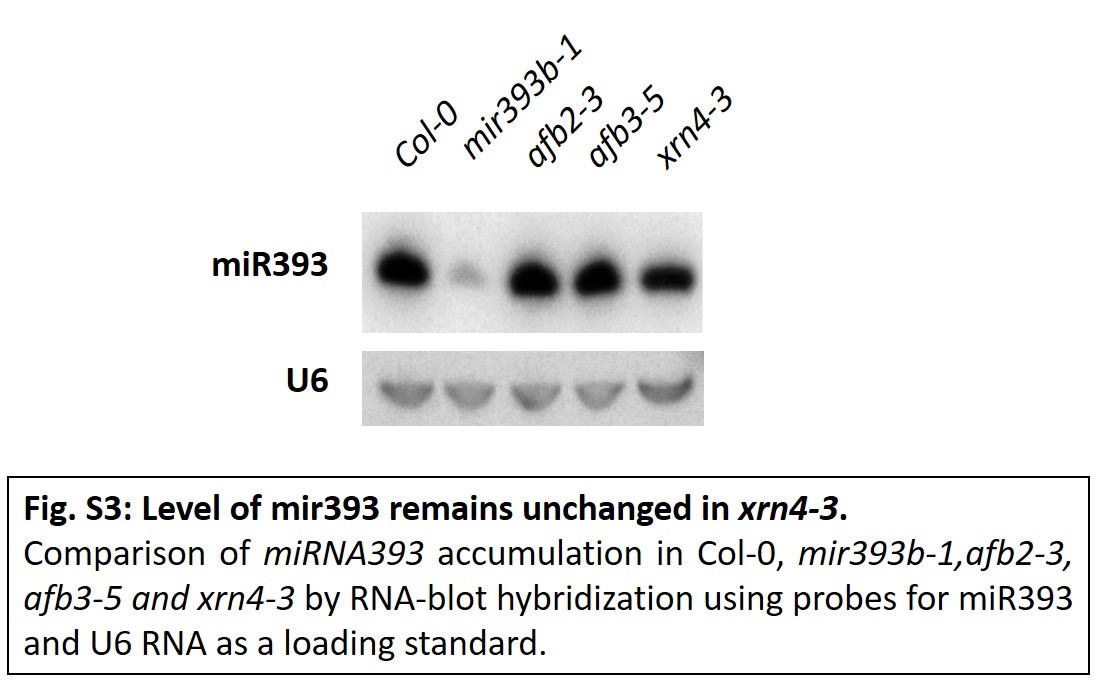

Supplement: Supplementary file 1 [file genes-09-00638-s001.zip › SF3.jpg]

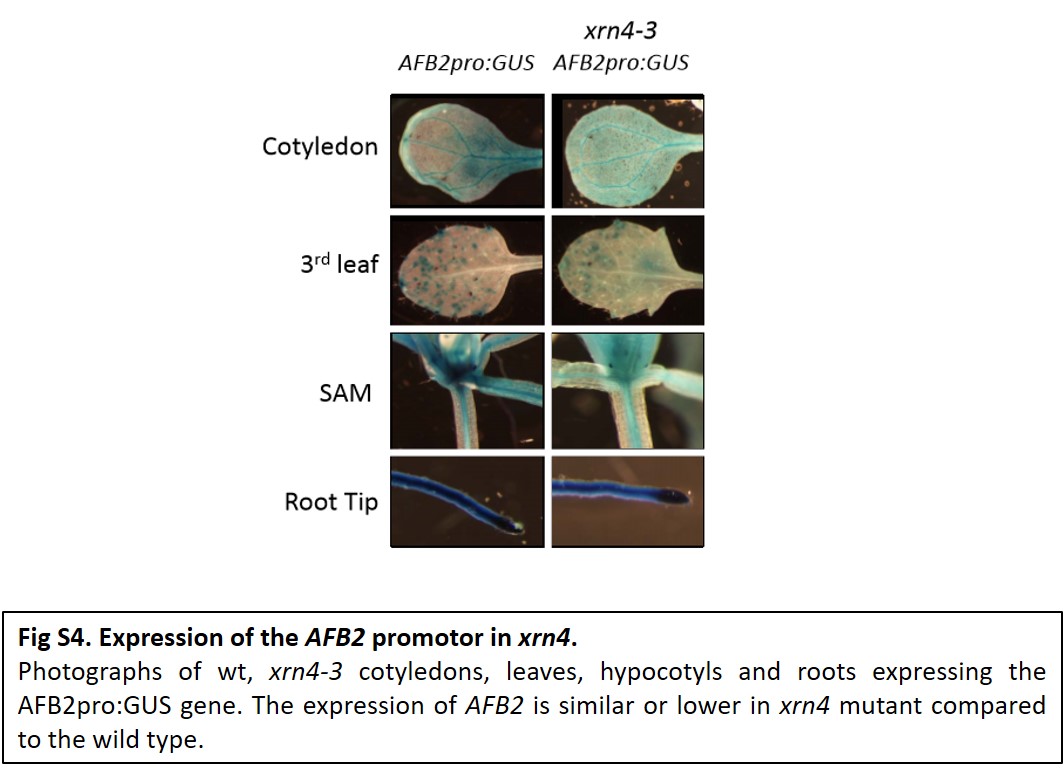

Supplement: Supplementary file 1 [file genes-09-00638-s001.zip › SF4.jpg]
